# Personality traits and social loafing among employees working in teams at small and medium enterprises: A cultural perspective data from emerging economies

**DOI:** 10.1016/j.dib.2022.108085

**Published:** 2022-03-23

**Authors:** Syed Asad Abbas Bokhari, Muhammad Aftab

**Affiliations:** a Ph.D. Candidate, Center of Convergence Security and eGovernance, Inha University, Nam-gu, Incheon 402-751, Korea; b Assistant Professor, International Graduate School, Namseoul University, Cheonan 31019, Korea

**Keywords:** Personality traits, social loafing, individualism, Culture, Big Five factory personality

## Abstract

The main objective of this research is to examine the association between personality traits such as conscientiousness and neuroticism and social loafing behavior of employees with moderating impact of individualistic behaviors. A multi correlational survey was used to investigate and analyze this study. The survey sample consisted of 241 supervisors and subordinates who attended a survey at manufacturing firms operating in three South Asian countries e.g., Pakistan, Bangladesh, and India. In the data analysis, a reliability and validity test were conducted and then correlation and regression analyses were used to investigate the results. Statistically substantial differences on some study variables were perceived vis-à-vis cultural traits. Correlation and regression verdicts exhibited that there was a substantial negative association between conscientiousness and social loafing, and a positive association between neuroticism and social loafing. Furthermore, this relationship between variables was strengthened when an individual behavior was included as moderator.

## Specifications Table

**Table:** 

Subject	Business, Management, and Decision Sciences: Organizational behavior and Human Resource Management
Specific subject area	“Impact of personality traits on work performance in different cultures of emerging economy's small and medium enterprises”
Type of data	Tables and Figures
How the data were acquired	Survey questionnaire was used to collect data for this research. The sample data for this study were collected from three countries and the firms functioning in the manufacturing of automobiles, textiles, food, metal, leather, paper, and petroleum, etc., in Pakistan Bangladesh, and India by picking similar teams with each other according to key variables. We congregated data from both supervisors and subordinates working together in a group. A total of 65 out of 355 supervisors were agreed to participate in the survey and filled and returned us their survey forms. A total of 930 subordinates worked under the supervision of these 65 supervisors. From these 930 subordinates, 176 returned their survey forms. We didn't find any missing value from both supervisor's and subordinates' forms. The questionnaires were provided to Managers through emails and requested them to fill and submit back in reply.
Data format	“Raw”
Description of data collection	We collected data using above given questionnaire and measured it on 5-point Likert-scale. After collecting data, we calculated average of four questions asked in each component to investigate our proposed hypothesis further. Higher average of conscientiousness denoted to more efficiency at workplace which is positive sign and have negative relationship with social loafing. On the other hand, Neuroticism is negative trait which has positive relationship with social loafing. “Social Loafing is negative attitude at work while working in groups or teams so higher number in SL means low work performance of the individual”.
Data source location	• Institution: Manufacturing Firms in different industries • Country: Pakistan, India, Bangladesh
Data accessibility	Repository name: Mendeley Data Data identification number: 10.17632/k3ym65vgdp.1 Direct URL to data: Personality Traits and Social Loafing, Moderating impact of Individualism as culture - Mendeley Data

## Value of the Data

• After completing our survey and study, data for personality traits especially conscientiousness and neuroticism, individualism, and social loafing is available for future researchers. Most importantly, this data is collected from 3 different countries of south Asian countries where culture is similar and numerous previous researchers have focused their studies on firms operating in South Asia. Scholars conducting research on human behavior and personality at workplace can use this data to get surprising results.
• We utilised 16 items to investigate our four variables and those items were adapted from previous research. Future researcher working on their studies on cultural difference at workplace, working environment, big five personality traits and its impact on negative job performance of individuals on workplace can utilize this data for their future research. Moreover, researchers can attain and provide different opinions about individualism between a country culture and organizational culture using our data set.
• It is important to understand the context and situations where employees work under a manager so, researchers can use this data to investigate further contextual factors of personality traits on job performance of employees.

## 1 Data Description

While considering bout the gender, out of 65 supervisors, 16 (24%) were females and 49 (76%) were males, however among 176 subordinates, 49 (28%) were females and 127 (72%) were male. The age of supervisors and subordinates was ranging from 18 to 55 years, and education level was measure from high school to master's degree for both supervisors and subordinates in both genders. Segregation of demographic individualities according to countries is given in Table 1.

**Table 1:** Demographic individualities by country.

	PK (%)	BD (%)	IN (%)
**Gender**			
Male	69 (73%)	47 (72%)	60 (73%)
Female	25 (27%)	18 (28%)	22 (27%)
**Age**			
18 to 34	38%	29%	36%
35 to 45	36%	45%	39%
46 to 55	26%	26%	25%
**Education**			
High School	8%	5%	4%
Intermediate	45%	48%	49%
Bachelor	38%	40%	39%
Master	9%	7%	8%

*Note. N = 241. PK = Pakistan; BD = Bangladesh; IN = India.

### 1.1 Data measurements

We utilized three survey forms to estimate our constructs in this research. One survey form was sent to employees involved in the working teams and the other two forms were accomplished by the participating supervisors. Employees were requested to fill the survey form by keeping in view task visibility, personality dimension, and social loafing. We also added a section for demographic characteristics such as gender, age, group size, satisfaction, and tenure of members with the group. Supervisors, in one of the surveys, were requested to fill about the questions concerning their collaboration level with their subordinate group. The second survey form comprised of two dimensions; demographics and social loafing scale.

As mentioned earlier, it should be guaranteed that supervisors have provided the chance to witness their subordinates while working through their time and they have spent time together with them to demonstrate that social loafing data collected from a supervisor is dependable. This collected data from the survey was evaluated by asking the supervisor numerous demographic questions. Furthermore, a task visibility scale was delivered to the supervisors to evaluate whether the efforts made by an individual member in the group were distinguishable. We modified the team leader's insights of task visibility scale from [1], which comprised of 6 items.

 **Social Loafing Scale:** To measure the social loafing of respective individual subordinates, we established a scale for supervisors by adapting questionnaire from previous research [1]. Supervisors were requested to respond to the questions about each of their assistants and to rate each member in the group independently on the scale items. The answers from supervisors were investigated by Five-Point Likert Scale shifting from 1 “strongly disagree” to 5 “strongly agree”. We advanced this social loafing scale after the revision process. We attained four supplementary items in the survey by gaining information from an organized interview with 5 supervisors from different companies. Henceforth, 04 items were involved in the final version of the scale.
 **Demographics:** demographics of supervisors were evaluated by asking the questions regarding their age, gender, education level, total job experience, tenure, duration of the existence of the current group, duration of supervision with the current group, total number of group members, duration of supervision with their group, their satisfaction level with the group, and whether the supervisor has administered another group before.
To measure Individualism, we utilized 4 item factors which were the degree to feel bother from other people at work while not being handy person, to helping others at work and being hurt if ignore from others, to involve in caring to help others in their personal needs, and the degree to involve others needs and feelings while deciding at workplace.
 **Personality Traits:** We appraised the personality traits of subordinates/group/ employees/ members with an alteration form of the Big Five with a list of 44 adjectives [2]. The group affiliates were asked to scale themselves with the personality features in the survey conferring to their characteristics. Replies from subordinates were analyzed by Five-Point Likert Scale fluctuating from 1 “strongly disagree” to 5 “strongly agree”. Conscientiousness was assessed by 4 substances and neuroticism were assessed by 4 objects in the survey questionnaire.
We used the degree of handling staff smoothly and know how to get things done, degree to keep promises and do more than what is expected, scale to work hard and follow through plan, and the degree to shrink duties and exact in work to measure conscientiousness. Further, we utilized 4 substantial items to measure neuroticism which were: the degree to inclined to get oneself all worked up over nothing and easily embarrassed in social situation, to get worried unnecessarily over things that may happen and felt to go for a long-holidays, to often wake up sweating after bad dreams and blush more often than usual people do, and the degree to be anxious about something or somebody most of the time and feelings that one cannot overcome difficulties.
 **Control Variables:** Perceived satisfaction of Supervisor with his groups is inconstant that might be associated with social loafing. Various previous scholars found an inconsistent negative relationship between task visibility and social loafing [1,^3^,4]. Henceforth, gender, age, and educational variables that are highly correlated with dependent variables should be utilized as control variables.

## 2 Experimental Design, Materials and Methods

The data sample collected for this research was analyzed utilizing SPSS and SEM software and we used multi-regression analysis to test our hypothesis [5]. Pearson correlations amongst all social loafing procedures were used to examine convergent validity. Correlation coefficients were considered between personality traits and social loafing measures. Finally, all relevant correlation coefficients were calculated between independent variables, dependent variables, and moderators to investigate our hypothesized relationships.

**Fig. 1:**
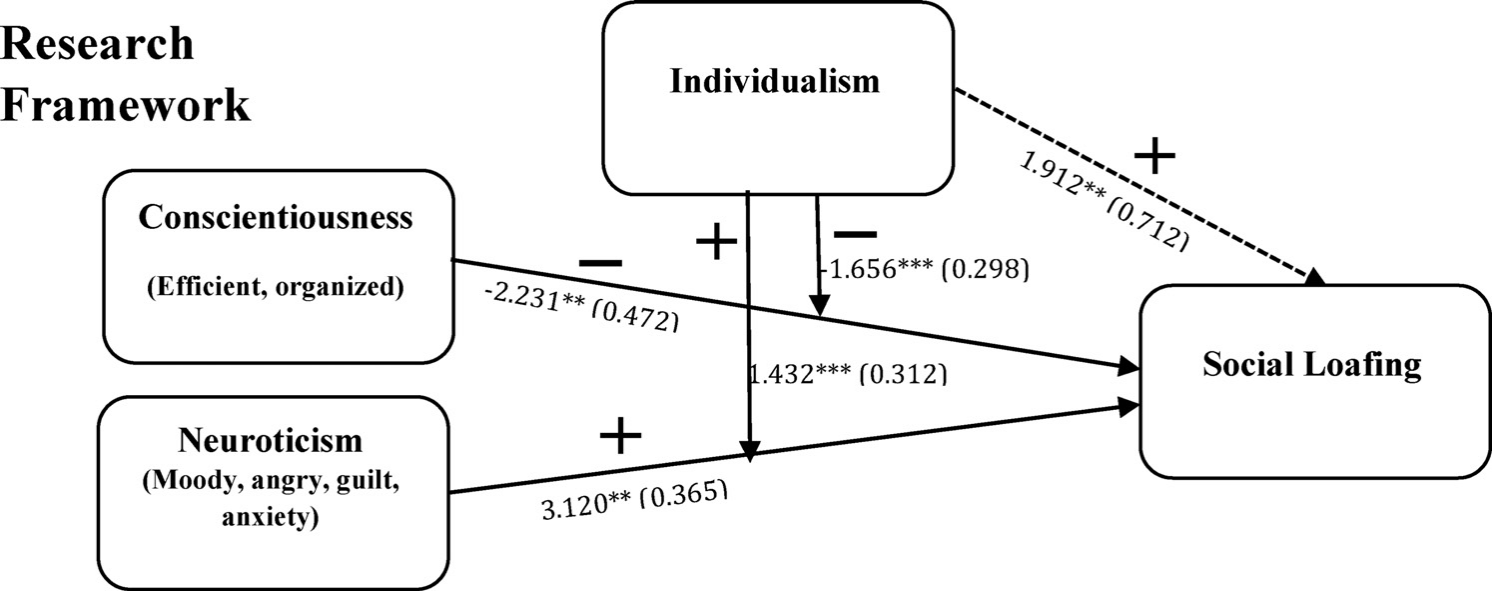
Link between conscientiousness, and neuroticism (personality traits) and social loafing with moderation impact of individualism.

Fig. 1 displays our hypothesized research framework where our independent variables such as conscientiousness and neuroticism are considered as personality traits of employees working in groups, individualism as moderators, and social loafing is considered as the dependent variable which denotes lower job performance. Our research model demonstrates that conscientiousness has an insignificant negative relationship with social loafing, conversely, neuroticism has a significant positive relationship with social loafing. Furthermore, when individualism is included as a moderator between both independent variable personality traits and dependent variable social loafing, the association between conscientiousness became weakened with social loafing, and the affiliation between neuroticism and social loafing became strengthened. Observing Fig. 1, we established the following research framework equation of our hypothesis:

(i) SL=β0+β1CNTCS+β2CNTCSxIND+β3CONTROLS+ϵi

(ii) SL=β0+β1NRTCM+β2NTRCMxIND+β3CONTROLS+ϵi

Where ‘CNTCS’ signifies conscientiousness (positive personality trait), ‘NRTCM’ discovers neuroticism (negative personality trait), ‘IND’ indicates Individualism, ‘SL’ implies social loafing (reduced job performance of individual person working in a group), ϵi represents error term is variable, β0 describes intercept while β1,β2, and β3 designate coefficients of variables.

## Ethics Statements

We confirm that the data was collected using social media platforms hence, ethics governing body name and protocol number is not applicable. We didn't need ethical approval from any appropriate institutional review boards and ethics committees. Furthermore, it is confirmed that informed consent of all participants has been obtained.

## CRediT Author Statement

**Syed Asad Abbas Bokhari:** Conceptualization, Writing – original draft preparation, Writing – review & editing, Methodology, Investigation, Resources; **Muhammad Aftab:** Conceptualization, Writing – review & editing, Methodology, Resources.

## Declaration of Competing Interest

The authors declare that they have no known competing financial interests or personal relationships that could have appeared to influence the work reported in this paper.

## Data Availability

Personality Traits and Social Loafing, Moderating impact of Individualism as culture (Original data) (Data collected through Social Media).

## Appendix Supplementary materials

Image, application 1
