# Supplementary material for: Personality traits and social loafing among employees working in teams at small and medium enterprises: A cultural perspective data from emerging economies
Source: Data Brief. 2022 Mar 23;42:108085. doi: 10.1016/j.dib.2022.108085 (PMC8980617; doi:10.1016/j.dib.2022.108085)
Supplement: Supplementary file 1 [file mmc1.docx]

**Questionnaire to Collect Data**

**Conscientiousness**

1. I handle tasks smoothly and I know how to get things done.
2. I keep my promises and I do more than what's expected of me.
3. I work hard and I follow through with my plans.
4. I shirk my duties and I am exacting in my work.

**Neuroticism**

1. Are you inclined to get yourself all worked up over nothing and easily embarrassed in a social situation?
2. Do you worry unnecessarily over things that might happen and ever felt you needed to take a very long holiday?
3. Do you often wake up sweating after having a bad dream and blush more often than most people?
4. Are you anxious about something or somebody most of the time and sometimes feel that you have so many difficulties that you cannot possibly overcome them?

**Individualism**

1. It bothers me when other people neglect my needs, and I don't consider myself to be a particularly helpful person
2. I don't especially enjoy giving others aid and when I have a need that others ignore, I'm hurt
3. I believe it's best not to get involved in taking care of other people's personal needs.
4. When deciding, I take other people's needs and feelings into account and I often go out of my way to help another person

**Social Loafing**

1. In a team, I am not indispensable, and I will try as hard as I can
2. In a team, I will contribute less than I should, and it is okay even if I do not do my share
3. In a team, I will actively participate in the discussion and contribute and given my abilities, I will do the best I can
4. In a team, it does not matter whether or not I try my best
